# Interactions between patterns of multimorbidity and functional status among hospitalized older patients: a novel approach using cluster analysis and association rule mining

**DOI:** 10.1186/s12967-024-05444-9

**Published:** 2024-07-18

**Authors:** Francesco Piacenza, Mirko Di Rosa, Luca Soraci, Alberto Montesanto, Andrea Corsonello, Antonio Cherubini, Paolo Fabbietti, Mauro Provinciali, Rosamaria Lisa, Anna Rita Bonfigli, Elvira Filicetti, Giada Ida Greco, Lucia Muglia, Fabrizia Lattanzio, Mara Volpentesta, Leonardo Biscetti

**Affiliations:** 1 Unit of Advanced Technology of Aging Research, IRCCS INRCA, Ancona, Italy; 2 Centre for Biostatistics and Applied Geriatric Clinical Epidemiology, IRCCS INRCA, Ancona, Cosenza, Italy; 3 Unit of Geriatric Medicine, IRCSS INRCA, Cosenza, Italy; 4 Department of Biology, Ecology and Earth Sciences, University of Calabria, Cosenza, Italy; 5 Department of Pharmacy, Health and Nutritional Sciences, University of Calabria, Rende, Italy; 6 Geriatria, Accettazione Geriatrica e Centro di Ricerca Per L’invecchiamento, IRCCS INRCA, Ancona, Italy; 7 Scientific Direction, IRCCS INRCA, Ancona, Italy; 8 Unit of Neurology, IRCCS INRCA, Ancona, Italy; 9 Department of Clinical and Molecular Sciences, Università politecnica delle Marche, Ancona, Italy

**Keywords:** Multimorbidity, Functional status, Association rule mining, Cluster analysis, Older patients, Hospitalization

## Abstract

**Background:**

Multimorbidity (MM) is generally defined as the presence of 2 or more chronic diseases in the same patient and seems to be frequently associated with frailty and poor quality of life. However, the complex interplay between MM and functional status in hospitalized older patients has not been fully elucidated so far. Here, we implemented a 2-step approach, combining cluster analysis and association rule mining to explore how patterns of MM and disease associations change as a function of disability.

**Methods:**

This retrospective cohort study included 3366 hospitalized older patients discharged from acute care units of Ancona and Cosenza sites of Italian National Institute on Aging (INRCA-IRCCS) between 2011 and 2017. Cluster analysis and association rule mining (ARM) were used to explore patterns of MM and disease associations in the whole population and after stratifying by dependency in activities of daily living (ADL) at discharge. Sensitivity analyses in men and women were conducted to test for robustness of study findings.

**Results:**

Out of 3366 included patients, 78% were multimorbid. According to functional status, 22.2% of patients had no disability in ADL (functionally independent group), 22.7% had 1 ADL dependency (mildly dependent group), and 57.4% 2 or more ADL impaired (moderately-severely dependent group). Two main MM clusters were identified in the whole general population and in single ADL groups. ARM revealed interesting within-cluster disease associations, characterized by high lift and confidence. Specifically, in the functionally independent group, the most significant ones involved atrial fibrillation (AF)-anemia and chronic kidney disease (CKD) (lift = 2.32), followed by coronary artery disease (CAD)-AF and heart failure (HF) (lift = 2.29); in patients with moderate-severe ADL disability, the most significant ARM involved CAD-HF and AF (lift = 1.97), thyroid dysfunction and AF (lift = 1.75), cerebrovascular disease (CVD)-CAD and AF (lift = 1.55), and hypertension-anemia and CKD (lift = 1.43).

**Conclusions:**

Hospitalized older patients have high rates of MM and functional impairment. Combining cluster analysis to ARM may assist physicians in discovering unexpected disease associations in patients with different ADL status. This could be relevant in the view of individuating personalized diagnostic and therapeutic approaches, according to the modern principles of precision medicine.

**Supplementary Information:**

The online version contains supplementary material available at 10.1186/s12967-024-05444-9.

## Introduction

The progressive aging of the population in developed countries has been accompanied by a dramatic increase in the prevalence of multimorbidity (MM), characterized by the coexistence of 2 or more chronic diseases in the same individual [1, 2]. This condition represents a major challenge for healthcare systems worldwide [3, 4] and is becoming more and more common among older people as longevity increases [5–8]. Compared with older people with a single chronic condition, those with MM have an increased risk for becoming dependent on long-term care; MM, indeed, increases the risk for functional disability, poor quality of life, high mortality risk, and high healthcare utilization and costs [4, 9–12].

The interplay between functional impairment and MM is complex and influenced by the existence of bidirectional relationships [13, 14]. Indeed, on one hand the accumulation of chronic diseases may lead to functional impairment [15, 16]; on the other hand, functional decline has been reported to be associated with increased risk to develop multiple chronic diseases, thus enhancing the severity and the burden of MM [15, 17]. Furthermore, patterns of MM may change as a function of physical performance, as reported by Corsonello et al. in a cohort of older individuals attending outpatient services throughout Europe [18]. Some studies also suggested that functional deterioration might be a stronger predictor of poor outcomes compared with MM among older hospitalized patients [17, 19–21].

However, in patients with MM, specific combinations of diseases seem to have different effects on functional ability [22–26]; indeed, cardiovascular diseases/anemia/dementia, sensory impairment/cancer and respiratory/musculoskeletal patterns were reported to be associated with decline in both basic activities of daily living (BADL) and instrumental activities of daily living (IADL), whereas metabolic disorders and sleep disturbances with IADL impairment only [23]. Similarly, in the longitudinal cohort examined by Jackson et al. [26], cardiovascular and neurological disease patterns were associated with ADL and IADL decline, respectively. When considering specific clusters involving highly disabling diseases such as neurological disorders, disease severity has been associated with increased functional dependency [23, 27, 28]. In this regard, dementia at early stages and other neurological and mental diseases are mainly associated with decline in IADL, while advanced dementia by definition implies impairment in both BADL and IADL [23, 27, 28].

To disentangle the complex interplay between functional impairment and MM, it is then necessary to implement an approach combining both quantitative and qualitative assessment. In this context, several studies in the last years explored MM by applying different methods (e.g. prevalence figures, conditional counts, logistic regression models, latent class analysis, cluster analysis or network analysis and other data mining techniques) [29–32] to explore disease associations in different populations [19, 33–38]. However, most studies have included community-dwelling or outpatient cohorts [18, 24–28, 32, 33, 39], while those on hospitalized older patients [19] were based on single algorithms and did not explore within-cluster associations of diseases.

Using a combination of cluster analysis and ARM techniques already proposed in community-dwelling older patients [33], we performed a retrospective cohort study to investigate the putative relationships between ADL functional status and patterns of MM in hospitalized older patients. We aimed at: (a) exploring patterns of MM and within-pattern disease associations in a cohort of hospitalized older patients; (b) verifying whether patterns of MM change after stratifying for ADL disability; (c) identifying microarchitectural within-pattern disease associations potentially contributing to ADL disability.

## Methods

### Study design and participants

The present investigation used data from the ReportAGE study, a large observational study on outcomes of older patients admitted to acute care hospitals of Italian National Institute on Aging (INRCA-IRCCS). Data collected in 3 acute care wards of geriatric medicine in 2 research hospitals were used. Briefly, all patients consecutively admitted to these wards from September 2011 to December 2017 able to give informed consent were asked to participate in the present study. After obtaining written informed consent, all patients underwent a comprehensive geriatric assessment (CGA) by Inter-RAI Minimum Data Set acute care (MDS-AC) [40] conducted both at the time of hospitalization and at discharge. Criteria for inclusion were age > 65 years, length of stay more than 24 h, and signed informed consent. Participating physicians and nurses were specifically trained before starting recruitment, as previously described [41]. Overall, 3890 patients were successfully enrolled during the study period. From this sample, 443 people died during hospitalization and 81 subjects with missing data for ADL were excluded, thus leaving a final sample of 3,366 patients to be included in the analysis. The Ethics Committee of the Italian National Center on Aging approved the study protocol (Trial Registration no. NCT01397682). The study was performed in strict accordance with Helsinki Declaration.

The information collected at the patient’s discharge were used to build the exposure variables. Physical performance was assessed by evaluating the number of ADLs impaired at discharge, where impairment was defined as having need of intensive assistance or total dependency in performing each activity. A total of 7 ADL activities was evaluated: bathing, personal hygiene, toilet use, locomotion on unit, transfer, bed mobility and eating. According to ADL functioning, patients were further categorized in 3 groups: (a) patients without ADL disability (functionally independent group); (b) patients with 1 ADL dependency (mildly dependent group); and (c) patients with 2 or more ADL dependencies (moderately-severely dependent group). Discharge diagnoses were coded by the physician by using International Classification of Diseases, Ninth Revision, Clinical Modification (ICD-9 CM) codes. For each individual diagnosis, a dichotomic analytic variable was calculated. For this study, only diagnoses with prevalence greater than or equal to 5% were included in the analysis: hypertension, atrial fibrillation (AF), heart failure (HF), coronary artery disease (CAD), cerebrovascular disease (CVD), chronic kidney disease (CKD), chronic obstructive pulmonary disease (COPD), diabetes, anemia, dementia, cancer, prostate hypertrophy, Parkinson’s disease, thyroid dysfunction, osteoporosis.

### Statistical analysis

First, descriptive analysis of the study population before and after stratification by ADL status was provided. Data were reported as mean and standard deviation (SD) for continuous variables with normal distribution (assessed by Shapiro–Wilk test) and number and percentages (%) for categorical ones. The ANOVA test and the Chi-square test were performed to compare continuous and categorical variables across the three ADL groups, as appropriate. *P* values less than 0.05 (two-sided) were considered statistically significant.

Given the complexity of the study population, we used a 3-step approach including co-occurrence matrices, cluster analysis and ARM techniques to investigate the interplay between MM and functional status. First, we created co-occurrence matrices to investigate the most common pairwise disease associations in the overall study population, in men and women, and in distinct ADL groups; then, we employed a co-occurrence heatmap to depict the joint prevalence of disease pairs, representing the frequency with which pairs of diseases co-occur simultaneously. Second, as older patients are frequently characterized by complex disease interactions which go beyond pairwise associations, we applied hierarchical cluster analysis and ARM to explore such complex patterns; combination of ARM and cluster analysis is more sensitive than cluster analysis alone in detecting associations of less common diseases within clusters and overcome the limits of overall ARM alone in yielding important associations in highly complex population [42]. As ARM is very sensitive to the presence of diseases with low frequency, we included only 15 diseases with a prevalence of at least 5% of the study population, in order to avoid detecting spurious associations.

In brief, to analyse different patterns of associative multimorbidity without any a priori hypothesis, we used a hierarchical agglomerative clustering approach to create dendrograms identifying patterns of MM in the whole study population and after stratifying by ADL group. A binary distance measure (the Jaccard coefficient) was used to measure similarity and produce the distance matrix resulting in more distinctive clusters compared with other proximity measures. For dendrograms analysis we used the Ward method as previously reported [8] to find compact clusters and minimize the variance within clusters [9]. Furthermore, we performed ARM analysis to identify patterns and combinations that meet a minimum requirement for prevalence and at the same time occur much more frequently together than would be expected under statistical independence [43]. The optimal number of clusters has been defined by using visual inspection and the elbow method. ARM was conducted for each cluster of diseases identified by the hierarchical cluster analysis both in the overall population and in distinct ADL groups. We used 3 measurement ratios: support (how frequently the disease combinations appear in the data set), confidence (the conditional probability that a participant who has the antecedent disease will also have the consequent disease), and lift (the ratio of the observed support to that expected if the 2 events were independent). Lift measures the importance of a rule within an ARM and, therefore, was considered the main measure of significance in the study. A lift of more than 1 indicates that the antecedent conditions and the consequent conditions appear more often together than expected, which can be interpreted as the antecedent having a positive effect on occurrence of the consequent. A lift of less than 1 indicates that the antecedent and consequent conditions appear less often than expected, which means that the occurrence of the antecedent has a negative effect on the occurrence of the consequent, and a lift close to 1 indicates that the antecedent and the consequent conditions occur as often together as expected, meaning that occurrence of the antecedent has little or no effect on occurrence of the consequent conditions. Hence, the higher the lift, the higher the chance of co-occurrence of the consequent with the antecedent and the more significant the association. In order to obtain at least 10 rules for each cluster and compare the results (disease combinations by lift) among the 3 ADL groups (functionally independent, mildly dependent and moderately-severely dependent group), support and confidence were set 0.01 and 0.3, respectively. Data are presented using summary tables of association rules and graphical visualizations showing patterns of diseases. In order to facilitate a clear visualization of the associations between the diseases, a maximum of 15 rules were displayed for each cluster. To evaluate qualitative and quantitative ARM results without an antecedent subdivision by hierarchical clustering, ARM analysis was repeated in the whole study population and ADL groups without considering clusters. Finally, to evaluate the influence of sex on the observed associations, sensitivity analyses were conducted in men and women separately. Statistical analysis was carried out by Stata software version14 (StataCorp LLC) and R (version 4.6.0, R Development Core Team, 2014).

## Results

### Demographic characteristics of the whole cohort

Of the 3,366 participants (mean age = 85.7 ± 6.2 years), 2,012 (60%) were women. Mean age increased across ADL groups, from 82.3 ± 6.2 years in the functionally independent group to 85.9 ± 5.5 years in the mildly dependent one and 87.6 ± 5.7 years in the moderately-severely dependent group (p < 0.001). Similarly, the corresponding figures for the prevalence of women across the 3 groups were 49%, 59% and 66%, respectively (p < 0.001).

### Distribution of diseases in the overall study population and ADL groups

Among all the 40 diseases assessed, only 15 were present at least in the 5% of the study population and thus considered in this study (Table 1). A total of 199 participants (6%) had no chronic conditions, 547 (16%) had a single chronic condition, and 2,620 (78%) were affected by 2 or more chronic conditions. Hypertension, dementia, and CVD were the 3 most common chronic diseases in the whole population, with a prevalence of 44%, 27%, and 25%, respectively (Table 1). According to ADL status at discharge, 27.4% of patients had no ADL dependency, 22.2% had dependency in 1 ADL, while 50.4% were disabled in 2 or more ADLs. The disease prevalence was significantly different among ADL groups (Table 1). Specifically, neurodegenerative and CVD increased in prevalence in patients with increasing ADL impairment, thus reaching their highest prevalence in patients with 2 or more ADL impaired (moderately-severely dependent group); other diseases, like CKD and CAD had the highest prevalence in the mildly dependent group, compared to the other two groups. Finally, some medical conditions including hypertension, diabetes mellitus, COPD and prostate hypertrophy had a significantly lower prevalence in more disabled patients. The disease count was also significantly different between ADL groups (Additional file 1).

**Table 1:** Disease distribution among ADL groups

	All (n = 3366)	ADL = 0 (n = 923) (a)	ADL = 1 (n = 747) (b)	ADL = 2 (n = 1696) (c)	P value
Hypertension	1481 (44%)	443 (48.0%)c	363 (48.6%)c	675 (39.8%)ab	< 0.001
Dementia	919 (27.3%)	49 (5.3%)bc	150 (20.1%)ac	720 (42.5%)ab	< 0.001
CVD	850 (25.3%)	170 (18.4%)bc	194 (26.0%)a	486 (28.7%)a	< 0.001
CKD	835 (24.8%)	203 (22.0%)b	212 (28.4%)a	420 (24.8%)	< 0.001
Anemia	848 (25.2%)	220 (23.8%)	180 (24.1%)	448 (26.4%)	0.987
COPD	699 (20.8%)	215 (23.3%)c	179 (24.0%)c	305 (18.0%)ab	< 0.001
AF	695 (20.6%)	183 (19.8%)	164 (22.2%)	348 (20.5%)	0.986
Diabetes	645 (19.2%)	190 (20.6%)	160 (21.1%)	295 (17.4%)	< 0.05
HF	534 (15.9%)	155 (16.8%)	131 (17.5%)	248 (14.6%)	0.911
CAD	459 (13.6%)	138 (15%)c	132 (17.7%)c	189 (11.1%)ab	< 0.001
Cancer	297 (8.8%)	74 (8.0%)	66 (8.8%)	157 (9.3%)	0.786
Prostate hypertrophy	243 (7.2%)	92 (10.0%)c	57 (7.6%)	94 (5.5%)a	< 0.001
Parkinson’s disease	184 (5.5%)	10 (1.1%)bc	35 (4.7%)ac	139 (8.2%)ab	< 0.001
Thyroid dysfunction	204 (6.1%)	62 (6.7%)	53 (7.1%)	89 (5.2%)	0.998
Osteoporosis	190 (5.6%)	52 (5.6%)	57 (7.6%)c	81 (4.8%)b	< 0.05

AF: atrial fibrillation; CAD: coronary artery disease/ischemic cardiomyopathy; CKD: chronic kidney disease; COPD: chronic obstructive pulmonary disease; CVD: cerebrovascular disease; HF: heart failure

a: p < 0.05 when functionally dependent is the reference group

b: p < 0.05 when mildly dependent is the reference group

c: p < 0.05 when moderately-severely dependent is the reference group

### Pairwise associations between chronic diseases in the overall study population and ADL groups

The most common pairs of diseases in the overall study population (Additional Fig. S1) consisted of associations between hypertension-anemia, hypertension-CKD, hypertension-dementia, and AF-hypertension. Stratification by sex did not significantly modify observed disease associations (Additional Fig. S2, S3). Conversely, distribution of pairs of diseases across distinct ADL groups (Additional Fig. S4–6) significantly changed and followed that of single disease conditions: indeed, patients with moderate-severe functional impairment underwent a substantial increase of disease pairs including dementia and a slight increase of those pairs including CVD; similarly, patients with mild functional impairment were characterized by a significant increase in the prevalence of pairs including CKD and COPD.

### Cluster analysis and ARM in the overall study population and ADL groups

**Fig. 1:**
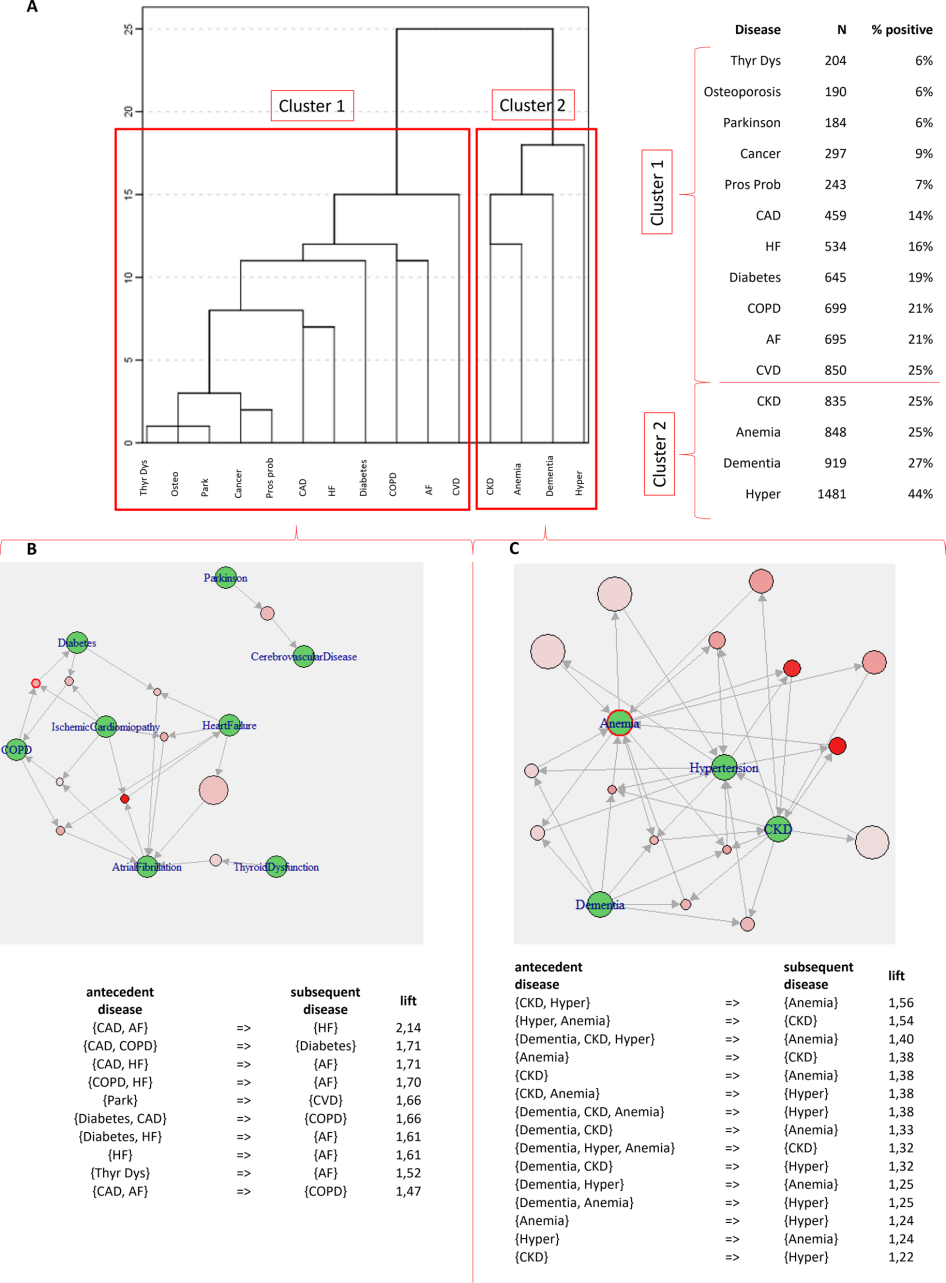
Patterns of MM by cluster analysis & ARM in the whole population. **A** Aggregation of diseases according to hierarchical cluster analysis; prevalence of disease contributing to each cluster is also reported; **B** ARM showing the most significant rules in cluster 1 in the whole population; **C** ARM showing the most significant rules in cluster 2 in the whole population. AF: atrial fibrillation; CAD: coronary artery disease/ischemic cardiomyopathy; CKD: chronic kidney disease; COPD: chronic obstructive pulmonary disease; CVD: cerebrovascular disease; HF: heart failure

By analyzing the increase in total within-cluster sum of squares, two main clusters of patients in the whole study population were identified (Fig. 1). Cluster 1 resulted to be very large and included cardiovascular diseases (AF, CAD, HF), thyroid dysfunction, COPD, diabetes, CVD, cancer, osteoporosis, Parkinson’s disease, and prostate hypertrophy; cluster 2 was instead made of CKD, anemia, dementia, and hypertension (Fig. 1A). Rules extracted through ARM were different in the 2 clusters: in cluster 1 (total 10 rules), the strongest association was that between cardiovascular diseases and included CAD-AF (antecedent diseases) and HF (subsequent disease, lift = 2.14) (Fig. 1B); in cluster 2 (total 20 rules), the strongest ones were those between CKD-hypertension and anemia (lift = 1.56) and hypertension-anemia and CKD (lift = 1.54) (Fig. 1C). All significant rules in cluster 1 and 2 of the whole population were reported in Table 2.

**Table 2:** Association rules showing disease relationships in cluster 1 and 2 of the overall study population

Antecedent	Consequent	Lift	Support
General population, cluster 1, rules = 10			
CAD, AF	HF	2.14	41 (1.2%)
CAD, COPD	Diabetes	1.71	43 (1.3%)
CAD, HF	AF	1.71	41 (1.2%)
COPD, HF	AF	1.70	47 (1.4%)
Parkinson’s	CVD	1.66	77 (2.3%)
Diabetes, CAD	COPD	1.66	43 (1.3%)
Diabetes, HF	AF	1.61	35 (1.0%)
CHF	AF	1.60	177 (5.3%)
Thyroid dysfunction	AF	1.52	64 (1.9%)
CAD, AF	COPD	1.47	37 (1.1%)
General population, cluster 2, rules = 20			
CKD, hypertension	Anemia	1.56	177 (5.3%)
Hypertension, anemia	CKD	1.54	177 (5.3%)
Dementia, CKD, hypertension	Anemia	1.39	45 (1.3%)
Anemia	CKD	1.38	291 (8.6%)
CKD	Anemia	1.38	291 (8.6%)
CKD, anemia	Hypertension	1.38	177 (5.3%)
Dementia, CKD, anemia	Hypertension	1.38	45 (1.3%)
Dementia, CKD	Anemia	1.33	74 (2.2%)
Dementia, hypertension, anemia	CKD	1.32	45 (1.3%)
Dementia, CKD	Hypertension	1.32	128 (3.8%)
Dementia, hypertension	Anemia	1.33	74 (2.2%)
Dementia, hypertension, anemia	CKD	1.32	45 (1.3%)
Dementia, CKD	Hypertension	1.32	128 (3.8%)
Dementia, hypertension	Anemia	1.25	137 (4.1%)
Dementia, anemia	Hypertension	1.25	137 (4.1%)
Anemia	Hypertension	1.24	463 (13.8%)
Hypertension	Anemia	1.24	463 (13.8%)
CKD	Hypertension	1.22	449 (13.3%)
Hypertension	CKD	1.22	449 (13.3%)
Dementia	Hypertension	1.08	435 (12.9%)

AF: atrial fibrillation; CAD: coronary artery disease/ischemic cardiomyopathy; CKD: chronic kidney disease; COPD: chronic obstructive pulmonary disease; CVD: cerebrovascular disease; HF: heart failure

**Fig. 2:**
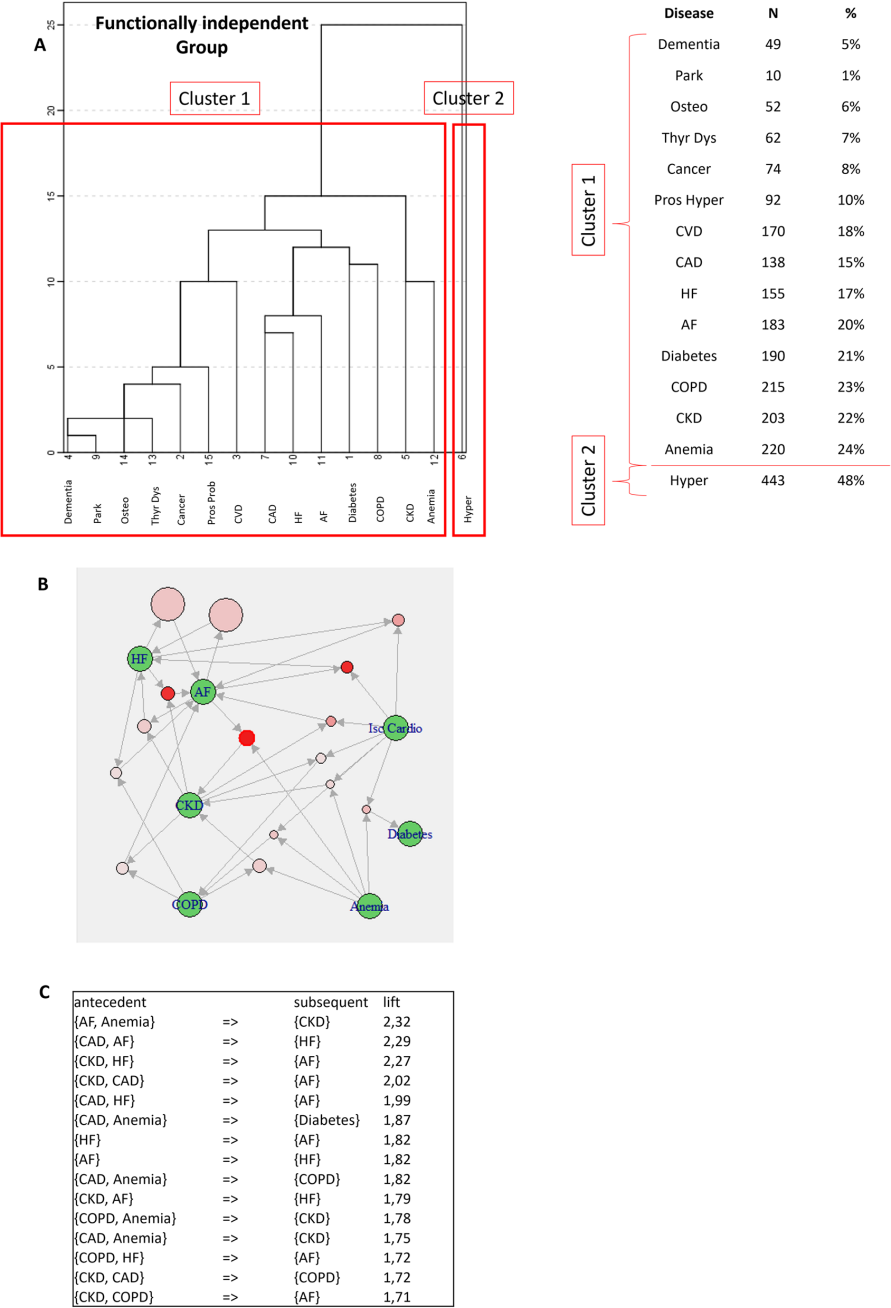
Patterns of MM by cluster analysis and ARM in the ADL = 0 group. **A** Aggregation of diseases according to hierarchical cluster analysis; prevalence of disease contributing to each cluster is also reported; **B** ARM graph showing the most significant rules in cluster 1 in the ADL = 0 group; **C** ARM table reporting the most significant rules in order of descending lift. AF: atrial fibrillation; CAD: coronary artery disease/ischemic cardiomyopathy; CKD: chronic kidney disease; COPD: chronic obstructive pulmonary disease; CVD: cerebrovascular disease; HF: heart failure

**Fig. 3:**
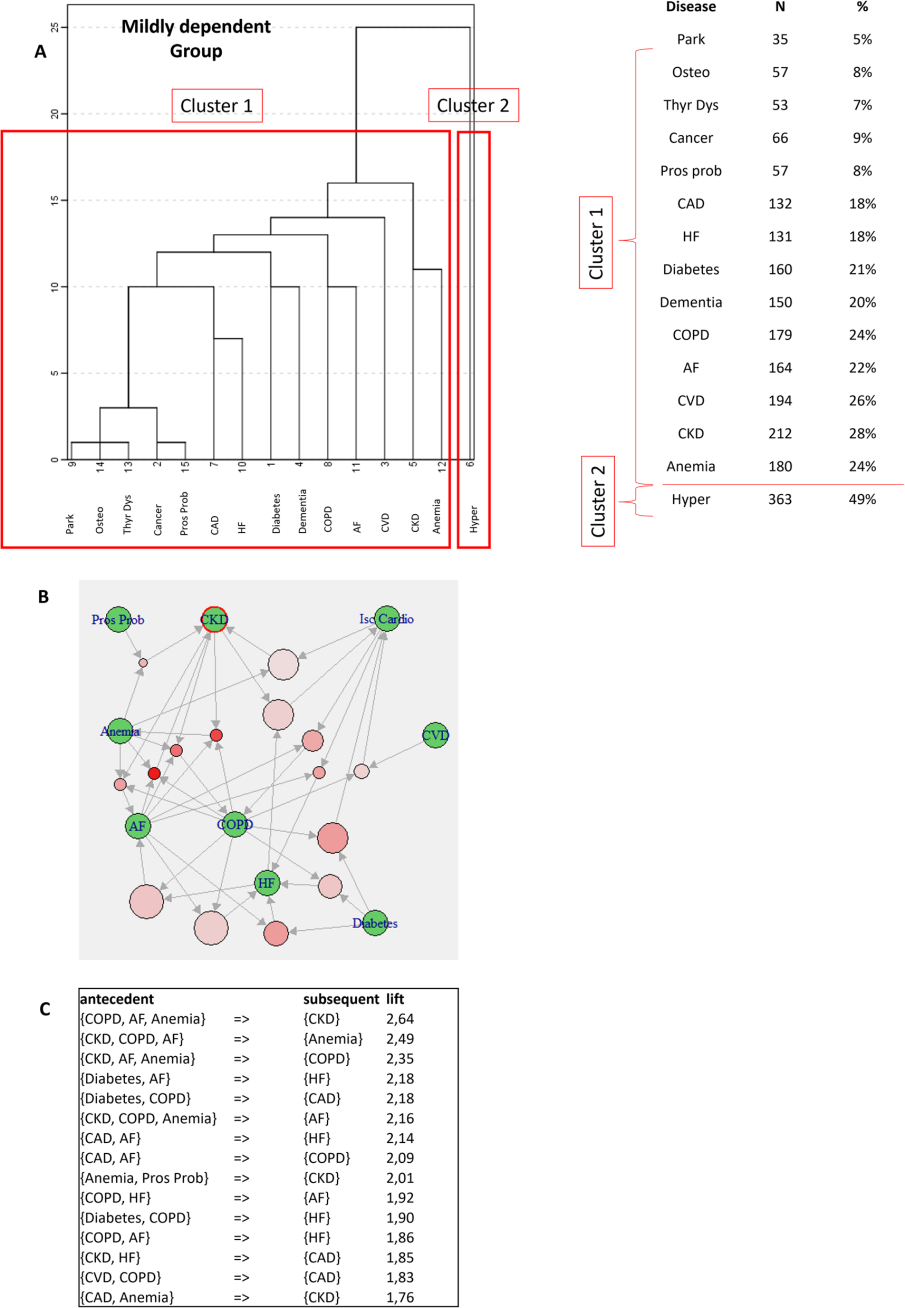
Patterns of MM by cluster analysis and ARM in the ADL = 1 group. **A** Aggregation of diseases according to hierarchical cluster analysis; prevalence of disease contributing to each cluster is also reported; **B** ARM graph showing the most significant rules in cluster 1 in the ADL = 1 group; **C** ARM table reporting the most significant rules in order of descending lift. AF: atrial fibrillation; CAD: coronary artery disease/ischemic cardiomyopathy; CKD: chronic kidney disease; COPD: chronic obstructive pulmonary disease; CVD: cerebrovascular disease; HF: heart failure

**Fig. 4:**
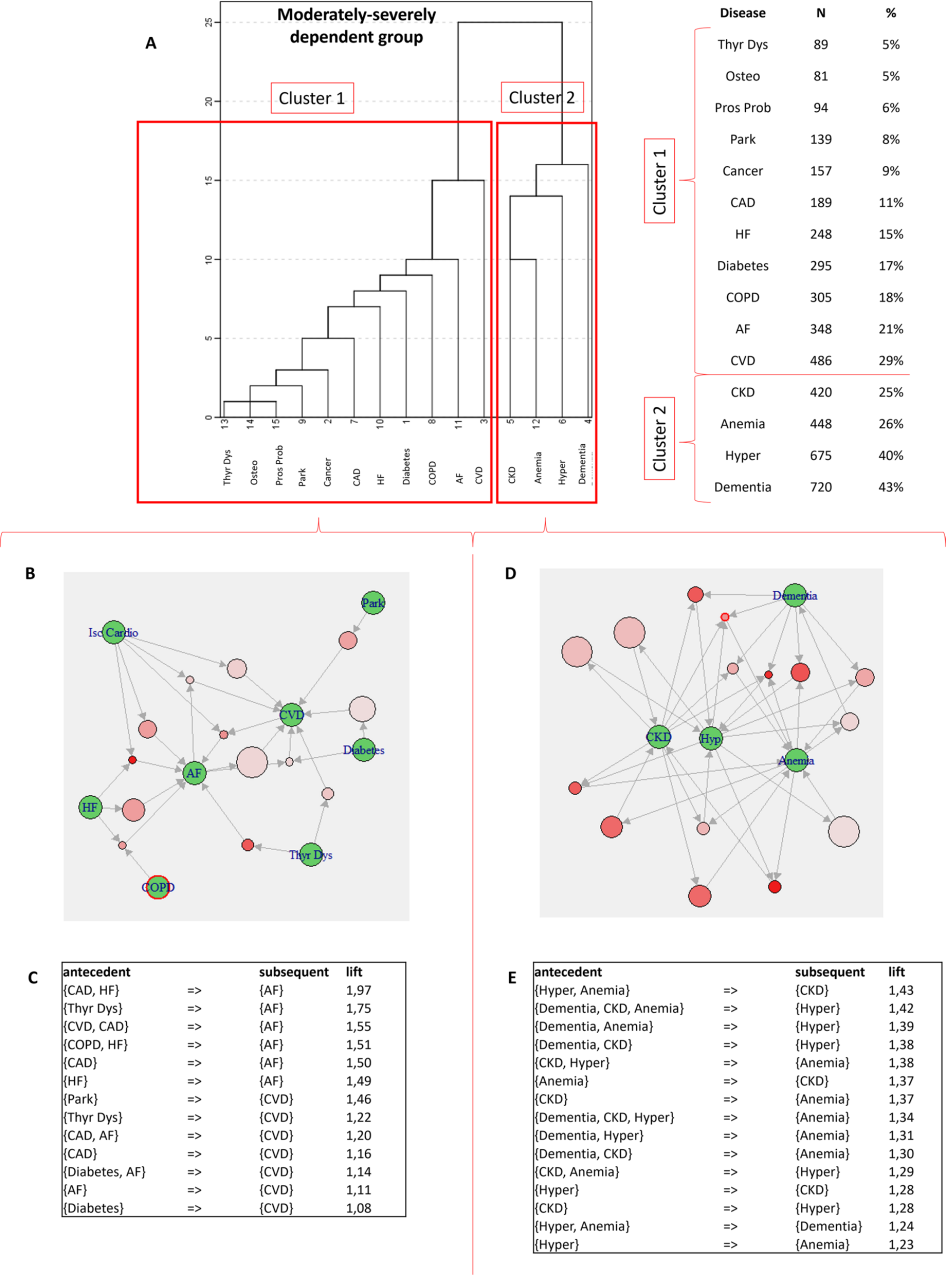
Patterns of MM by cluster analysis and ARM in the ADL = 2 group. **A** Aggregation of diseases according to hierarchical cluster analysis; prevalence of disease contributing to each cluster is also reported; **B** ARM graph showing the most significant rules in cluster 1 in the ADL = 2 group; panel **C **ARM table reporting the most significant rules in order of descending lift for cluster 1 in the ADL = 2 group; **D** ARM graph showing the most significant rules in cluster 2 in the ADL = 2 group; **E** ARM table reporting the most significant rules in order of descending lift for cluster 2 in the ADL = 2 group. AF: atrial fibrillation; CAD: coronary artery disease/ischemic cardiomyopathy; CKD: chronic kidney disease; COPD: chronic obstructive pulmonary disease; CVD: cerebrovascular disease; HF: heart failure

Stratification by ADL status provided clearly different results, depending on level of functional disability (Figs. 2–4). Among independent patients (functionally independent group), two main hierarchical clusters were identified, one including only hypertension and the other one including all other diseases considered in the analysis (Fig. 2A). Thus, ARM was applied only to cluster 1 (number of rules = 40) where the most significant associations were those between AF-anemia and CKD (lift = 2.32), CAD-AF and HF (lift = 2.29), CKD-HF and AF (lift = 2.27) and CKD-CAD and AF (lift = 2.02) (Fig. 2B and C). Hierarchical clusters obtained among patients with mild ADL dependency were similar to those of the functionally independent group; indeed, cluster 2 was always made by hypertension only, while cluster 1 included all the other diseases; however, central diseases of cluster 1 shifted from diabetes and cardiovascular diseases in functionally independent patients to COPD and dementia in mildly dependent ones (Fig. 3A). Use of ARM in this group provided interesting findings (Fig. 3B and C); in fact, the most significant associations observed in cluster 1 were those between COPD-atrial fibrillation-anemia and CKD (lift = 2.64), CKD-COPD-atrial fibrillation and anemia (lift = 2.49) and CKD-atrial fibrillation-anemia and COPD (lift = 2.35) (Fig. 3B and C). Finally, among patients with moderate-severe ADL dependency, MM clusters were equivalent to those found in the whole study population, as patients with moderate-severe ADL impaired represented the vast majority of the study population (Fig. 4A). In cluster 1, the most significant association rules involved CAD-HF and AF (lift = 1.97), thyroid dysfunction and AF (lift = 1.75), and CVD-CAD and AF (lift = 1.55) (Fig. 4B and C). In cluster 2, the most significant association rules involved hypertension-anemia and CKD (lift = 1.43), dementia-CKD-anemia and hypertension (lift = 1.42) and dementia-anemia and hypertension (lift = 1.39) (Fig. 4D and E). The list of significant association rules across all ADL groups are reported in Additional files 2, 3, and 4, respectively.

### Sensitivity analysis

Differences in the prevalence of chronic diseases between men and women are reported in Additional file 5. In brief, compared with men, women were characterized by a significantly higher prevalence of dementia, CKD, HF, thyroid dysfunction, and osteoporosis, and a lower prevalence of CVD, COPD, CAD, cancer, and Parkinson’s disease. Moderate-severe ADL impairment was slightly more common in women than men. Net of the differences in the prevalences of chronic disease, sex-stratified sensitivity analyses led to slightly distinct patterns in the male and female patients (Additional Fig. S7). Indeed, among men CKD, anemia, and hypertension aggregated in cluster 2, without dementia, which conversely gave a minor contribution to cluster 1. Among women, dementia and hypertension aggregated themselves into a smaller cluster 2, with all the other diseases including also CKD and anemia contributing to cluster 1 (Additional Figs. S7 and S8). After stratifying by ADL status, multimorbidity patterns of men were identical to those of the general population, and CKD, hypertension, anemia and dementia aggregated in cluster 2 in moderately-severely dependent patients; among women, the weight of dementia significantly contributed to cluster 2 along with hypertension, while CKD and anemia aggregated into cluster 1 in more disabled ADL patients (Additional Fig. S8). Sex-stratified ARM showed that most significant rules were similar to those of the general population; indeed, in cluster 1 and functionally independent patients, the highest lift was presented by the rule AF-anemia and CKD, CAD-AF and HF, and CKD-CAD and AF in both sexes; similar lifts were also shown in sex-stratified analysis for mildly dependent group, with more important associations represented by COPD-AF-anemia and CKD, CKD-COPD-AF and anemia. Conversely, in more disabled patients, the most significant rules in cluster 1 were those between CAD-AF and HF followed by thyroid dysfunction and AF in both sexes, as reported in the whole population.

## Discussion

Findings from the present study suggest multimorbidity significantly burdens older individuals discharged from acute care hospitals. Indeed, as already reported in previous studies [36, 38, 44, 45], the occurrence of a single disease without any co-occurring condition was rarely observed in older population. Specifically, in the present study, only 16% of the study cohort was affected by ≤ 1 chronic disease at the time of admission to hospital. Therefore, the great majority of included patients were multimorbid (i.e. with ≥ 2 chronic diseases). In our cohort, most patients had also functional disability: indeed, about 50% of them were moderately-severely dependent, having need of assistance in at least two activities of daily living, and 72.6% were impaired in at least one ADL. However, there was no evidence of a linear strong correlation between number of chronic diseases and degree of functional impairment. Simple count of diseases in fact may be often inadequate to capture the relationship between multimorbidity burden and functional disability; conversely, detailed assessment of disease pairs, MM clusters analysis and assessment of association rules among diseases within the whole cohort and within each MM cluster could improve diagnostic and prognostic stratification of elderly patients. To this regard, our study suggests that MM patterns may change as a function of ADL dependency when explored by cluster analysis or ARM. A sensitivity analysis after stratification by sex found a slight change of composition of MM patterns in both female and male subgroups in comparison with the whole cohort due to a higher prevalence of dementia among women compared to men and a lower prevalence of CKD in very disabled women than in men with similar functional status.

In the whole population, MM cluster analysis displayed a cluster including CKD, anemia, dementia, and hypertension, and a second one including all other chronic diseases. Interestingly, in stratified analysis such clustering could be retrieved only among patients with severe disability, but not among independent patients or those with intermediate degree of dependency. The fact that the cluster CKD-anemia-dementia-hypertension is evident only among patients with the highest functional impairment (ADL = 2) is explainable based on the very high impact of dementia on disability, since dementia causes by definition a variable degree of loss of autonomy in activities of daily living[46, 47]. However, all the diseases included in this cluster are known to share common pathophysiological pathways and to interplay in worsening functional status. Specifically, hypertension is one of the most important risk factors for CKD and CKD may cause and/or worsen hypertension, inducing a very dangerous vicious circle [48]. Furthermore, CKD and hypertension together may modify gut microbiota, thus potentially impairing the gut-brain axis, which is reported to be relevantly involved in the pathophysiology of cognitive impairment [49]. Moreover, epidemiological data supported a higher prevalence of dementia in patients with CKD compared to the general population [50]. Finally, CKD is a relevant cause of anemia [51] and their coexistence increases the risk for ADL dependency[52]; furthermore, both low hemoglobin and deteriorated kidney function are emerging as risk factors for cognitive impairment and dementia[53–55], and thus potentially ADL impairment. Therefore, the presence of a cluster including CKD, hypertension, anemia and dementia in our cohort and in patients with at least 2 disabilities is not surprising at all and these findings resulted to be very plausible from both a biological and epidemiological point of view.

With respect to ARM analysis, in the whole cohort, the strongest lift linked the association of CAD and AF as antecedent condition with HF as subsequent condition. This association was found to be strong also in various subgroups of patients with different functional status, suggesting that is relevant regardless of the degree of disability. Of note, the causal relationship among CAD, AF and HF is largely supported by the literature[56] and contributed to the highest lifts of ARM patterns including HF and AF in previous studies[35]. Stratification by functional status and cluster analysis significantly changed the strength and distribution of ARM patterns; in particular, the highest lifts characterised the combinations of AF-anemia and CKD in functionally independent patients and mildly dependent ones; most significant ARM patterns in highly dependent patients were represented by CAD-HF and AF in cluster 1, hypertension-anemia and CKD followed by dementia-CKD-anemia and hypertension in cluster 2. These findings confirm that aggregation of distinct disease conditions significantly change in function of ADL performance and many chronic diseases may interact each other to impact functional status; the association between AF-anemia and CKD in functionally independent patients is confirmed by previous 2-sample mendelian randomization study, showing a bidirectional causal relationship between anemia and cardiovascular diseases including AF [57]; furthermore, CKD and AF seem to be pathophysiologically interrelated, sharing several molecular mechanisms, including inflammation, aberrant activation of renin–angiotensin–aldosterone system and dysregulation of calcium homeostasis[58]. Previous studies have shown that AF, CKD, and HF were variably associated with functional impairment[18, 59, 60]; the coexistence of the three diseases in functionally independent patients may capture a segment of the geriatric population with mild disease severity and preserved functional performance; such patients still deserve accurate follow-up evaluations to promptly detect changes in the functional performance potentially due to chronic disease progression.

Interestingly, in the transition from no to mild functional impairment, COPD emerged as a diagnosis in most significant ARM patterns with COPD-AF-anemia and CKD being the most significant rule. Of note, COPD was reported to be independently associated to AF, probably due the combined effects of pulmonary hypertension, hypoxemia, hypercapnia, oxidative stress and inflammation, on the risk of atrial arrhythmia [61]. Additionally, some reports have suggested a potential link between COPD and anemia [62], as well as between COPD and CKD [63], but further studies will be needed to clarify the real clinical impact of the coexistence of these comorbidities on functional status. However, a previous study on community-dwelling patients has shown that patients with COPD experience only mild ADL disability and more severe IADL dependency [64], thus confirming results of our study. Furthermore, COPD may affect skeletal muscle metabolism and bioenergetics [65], thus potentially contributing to the onset of physical disability.

Finally, ARM patterns in most disabled group underlined the potential impact of cardiovascular diseases, and CVD in cluster 1, as well as depicting underlying relationships between CKD, anemia, and dementia in cluster 2; although CVD is recognized as a known risk factor for severe functional and cognitive impairment in the older population, the functional impact of cardiovascular diseases is highly variable depending on disease severity, type and population setting [60]. Indeed, among highly disabled patients, less impactful diseases like AF and hypertension were consistently replaced by CAD and HF in most significant rules; the impact of CAD and CVD on physical disability was recently confirmed by Kodera et al. in community-dwelling Japanese adults [66], among whom history of CVD and CAD substantially led to similar functional dependency; among patients belonging to cluster 2, the most significant rules involved the triad CKD-hypertension and anemia with or without dementia; this finding confirms that multimorbidity patterns of CKD change depending on functional status as already reported in community-dwelling older patients [18]; interestingly, aggregation of CKD with hypertension and anemia may intercept patients with moderate-severe CKD stage, who are at increased risk of severe ADL impairment and sarcopenia [67–70]. However, lack of information about disease severity did not allow us to confirm this hypothesis. As such, future studies including both prevalence and severity of chronic diseases are needed to disentangle the complex relationship between MM patterns and ADL disability.

Our study has several limitations: first, lack of information about the clinical severity of chronic diseases affecting older patients may have affected the change in MM patterns across ADL groups; second, the group of moderately-severely disabled patients included patients with a high range (from 2 to 8) of disabled ADL; this heterogeneity might have affected the reproducibility of the study findings; third, MM patterns’ overlapping between whole cohort and moderately-severely dependent patients may be due to the relative high prevalence of high ADL impairment in our study population with potential loss of information in less represented groups. Finally, as our study focused exclusively on hospitalized older patients, factors such as severity of illness, comorbidities, and treatment regimens unique to this setting may influence the observed outcomes. Furthermore, in the present study the patients’ enrolment was carried out in a Tertiary Geriatric Hospital specialized in the management of frail older subjects: this could lead to an overrepresentation of some categories of individuals, for instance those with dementia. Accordingly, results may not be generalizable to the entire older population and future research is needed to validate these findings in community-dwelling older individuals.

Faced to these limitations, our study has also some strengths. First, this is a real-word study potentially able to give useful insights about the clinical presentation in routine medical practice of older patients with different degrees of disability. Second, the sample size of the included cohort is relatively large (n = 3336). Third, sex-stratified sensitivity analyses conducted to test the robustness of study findings substantially confirmed our results, with only slight differences in disease distributions, mainly due to different prevalence of high-impact diseases, such as CKD and dementia.

In conclusion, our study suggests the existence of a link between different pattern of MM and functional impairment. Further prospective multicentric studies including prevalence and severity of chronic diseases will be needed to confirm these findings.

## Conclusions

Hospitalized older patients are a segment of geriatric population characterized by high MM burden and high functional disability. Interaction between the two may have significant impact on healthcare systems and pathways of care. Using a two-step statistical approach including cluster analysis and ARM may help physicians unveiling unexpected disease associations in more vulnerable patients and tailoring personalized approaches.

### Supplementary Information

Additional file 1:** Table S1.** Disease count in patients belonging to distinct ADL groups. ADL: activities of daily living. ^a^p < 0.05 when reference group is ADL = 0. ^b^p < 0.05 when reference group is ADL = 1. ^c^p < 0.05 when reference group is ADL = 2.

Additional file 2:** Table S2.** List of the 40 association rules showing disease relationships in the functionally independent group, cluster 1. AF: atrial fibrillation; CAD: coronary artery disease/ischemic cardiomyopathy; CKD: chronic kidney disease; COPD: chronic obstructive pulmonary disease; CVD: cerebrovascular disease; HF: heart failure.

Additional file 3:** Table S3.** List of the 73 association rules showing disease relationships in the mildly dependent group, cluster 1. AF: atrial fibrillation; CAD: coronary artery disease/ischemic cardiomyopathy; CKD: chronic kidney disease; COPD: chronic obstructive pulmonary disease; CVD: cerebrovascular disease; HF: heart failure.

Additional file 4:** Table S4.** List of the association rules showing disease relationships in cluster 1 and 2 of the moderately-severely dependent group. AF: atrial fibrillation; CAD: coronary artery disease/ischemic cardiomyopathy; CKD: chronic kidney disease; COPD: chronic obstructive pulmonary disease; CVD: cerebrovascular disease; HF: heart failure.

Additional file 5:** Table S5.** Differences in the prevalence of chronic diseases and ADL impairment between men and women. ADL: activity of daily living; AF: atrial fibrillation; CAD: coronary artery disease/ischemic cardiomyopathy; CKD: chronic kidney disease; COPD: chronic obstructive pulmonary disease; CVD: cerebrovascular disease; HF: heart failure.

Additional file 6:** Figure S1. **Heatmap representation of the co-occurrence of disease pairs in the whole study population. Each cell in the heatmap corresponds to a unique combination of diseases, with the color intensity indicating the strength of co-occurrence. Darker shades represent higher joint prevalence, highlighting disease pairs that commonly occur together.

Additional file 7:** Figure S2. **Heatmap representation of the co-occurrence of disease pairs in the whole study population (upper part) and among men (lower part). Each cell in the heatmap corresponds to a unique combination of diseases, with the color intensity indicating the strength of co-occurrence. Darker shades represent higher joint prevalence, highlighting disease pairs that commonly occur together.

Additional file 8:** Figure S3. **Heatmap showing the co-occurrence of disease pairs in the whole study population (upper part) and among women (lower part). Each cell in the heatmap corresponds to a unique combination of diseases, with the color intensity indicating the strength of co-occurrence. Darker shades represent higher joint prevalence, highlighting disease pairs that commonly occur together.

Additional file 9:** Figure S4. **Heatmap showing the co-occurrence of disease pairs in the whole study population (upper part) and among functionally independent (ADL = 0) patients (lower part) Each cell in the heatmap corresponds to a unique combination of diseases, with the color intensity indicating the strength of co-occurrence. Darker shades represent higher joint prevalence, highlighting disease pairs that commonly occur together.

Additional file 10:** Figure S5. **Heatmap showing the co-occurrence of disease pairs in the whole study population (upper part) and among mildly functionally dependent (ADL = 1) patients (lower part). Each cell in the heatmap corresponds to a unique combination of diseases, with the color intensity indicating the strength of co-occurrence. Darker shades represent higher joint prevalence, highlighting disease pairs that commonly occur together.

Additional file 11:** Figure S6. **Heatmap showing the co-occurrence of disease pairs in the whole study population (upper part) and among moderately-severely functionally dependent (ADL = 2) patients (lower part). Each cell in the heatmap corresponds to a unique combination of diseases, with the color intensity indicating the strength of co-occurrence. Darker shades represent higher joint prevalence, highlighting disease pairs that commonly occur together.

Additional file 12:** Figure S7. **Hierarchical cluster analysis showing MM patterns in women and men in the whole population. AF: atrial fibrillation; CAD: coronary artery disease/ischemic cardiomyopathy; CKD: chronic kidney disease; COPD: chronic obstructive pulmonary disease; CVD: cerebrovascular disease; HF: heart failure.

Additional file 13: **Figure S8. **Hierarchical cluster analysis showing MM patterns in women and men in the moderately-severely dependent group. AF: atrial fibrillation; CAD: coronary artery disease/ischemic cardiomyopathy; CKD: chronic kidney disease; COPD: chronic obstructive pulmonary disease; CVD: cerebrovascular disease; HF: heart failure.

## Data Availability

Anonymized data and code used in conducting the analyses will be made available upon request directed to the corresponding author.

## Abbreviations

ADL: Activities of daily living
AF: Atrial fibrillation
ARM: Association rule mining
CAD: Coronary artery disease/ischemic cardiomyopathy
CKD: Chronic kidney disease
COPD: Chronic obstructive pulmonary disease
CVD: Cerebrovascular disease
HF: Heart failure
MM: Multimorbidity
