# Supplementary material for: Interactions between patterns of multimorbidity and functional status among hospitalized older patients: a novel approach using cluster analysis and association rule mining
Source: J Transl Med. 2024 Jul 18;22:669. doi: 10.1186/s12967-024-05444-9 (PMC11264579; doi:10.1186/s12967-024-05444-9)
Supplement: Supplementary file 1 — Additional file 1: Table S1. Disease count in patients belonging to distinct ADL groups. ADL: activities of daily living. ap < 0.05 when reference group is ADL = 0. bp < 0.05 when reference group is ADL = 1. cp < 0.05 when reference group is ADL = 2. [file 12967_2024_5444_MOESM1_ESM.docx]

| **N diseases** | **Functionally**  **Independent (n=923)** | | **Mildly dependent**  **(n=747)** | | **Moderately-severely**  **dependent (n=1,696)** | | **P value** |
| --- | --- | --- | --- | --- | --- | --- | --- |
|  | **N** | **%** | **N** | **%** | **N** | **%** |  |
| **0** | 70^b^ | 8% | 33^a^ | 4% | 96 | 6% | <0.05 |
| **1** | 179^bc^ | 19% | 103^a^ | 14% | 265^a^ | 16% | <0.05 |
| **2** | 235 | 26% | 180 | 24% | 406 | 24% | ns |
| **3** | 245 | 27% | 184 | 25% | 430 | 25% | ns |
| **4** | 123^b^ | 13% | 148^a^ | 20% | 273 | 16% | <0.05 |
| **5** | 53^bc^ | 6% | 73^a^ | 10% | 144^a^ | 9% | <0.05 |
| **6** | 13^c^ | 1% | 21 | 3% | 60^a^ | 4% | <0.05 |
| **7** | 3 | 0.2% | 5 | 1% | 21 | 1% | ns |
| **8** | 2 | 0.3% | 0 | 0% | 1 | 0.05% | ns |
