# Supplementary material for: Interactions between patterns of multimorbidity and functional status among hospitalized older patients: a novel approach using cluster analysis and association rule mining
Source: J Transl Med. 2024 Jul 18;22:669. doi: 10.1186/s12967-024-05444-9 (PMC11264579; doi:10.1186/s12967-024-05444-9)
Supplement: Supplementary file 2 — Additional file 2: Table S2. List of the 40 association rules showing disease relationships in the functionally independent group, cluster 1. AF: atrial fibrillation; CAD: coronary artery disease/ischemic cardiomyopathy; CKD: chronic kidney disease; COPD: chronic obstructive pulmonary disease; CVD: cerebrovascular disease; HF: heart failure. [file 12967_2024_5444_MOESM2_ESM.docx]

| **Antecedent diseases** | **Consequent disease** | **Lift** | **Support** |
| --- | --- | --- | --- |
| AF, anemia | CKD | 2.32 | 23 (2.5%) |
| CAD, AF | HF | 2.29 | 15 (1.6%) |
| CKD, HF | AF | 2.27 | 18 (1.9%) |
| CKD, CAD | AF | 2.02 | 12 (1.3%) |
| CAD, HF | AF | 1.99 | 15 (1.6%) |
| CAD, anemia | Diabetes | 1.87 | 10 (1.1%) |
| HF | AF | 1.82 | 56 (6.1%) |
| AF | HF | 1.82 | 56 (6.1%) |
| CAD, anemia | COPD | 1.82 | 11 (1.2%) |
| CKD, AF | HF | 1.79 | 18 (1.9%) |
| COPD, anemia | CKD | 1.78 | 18 (1.9%) |
| CAD, anemia | CKD | 1.75 | 10 (1.1%) |
| COPD, HF | AF | 1.72 | 14 (1.5%) |
| CKD, CAD | COPD | 1.72 | 12 (1.3%) |
| CKD, COPD | AF | 1.72 | 17 (1.8%) |
| Diabetes, HF | AF | 1.68 | 10 (1.1%) |
| Diabetes, anemia | CKD | 1.65 | 16 (1.7%) |
| CKD, anemia | AF | 1.63 | 23 (2.5%) |
| CKD, CAD | Diabetes | 1.62 | 10 (1.1%) |
| CKD, AF | Anemia | 1.61 | 25 (2.5%) |
| COPD, anemia | Diabetes | 1.58 | 15 (1.6%) |
| Cancer | Anemia | 1.53 | 27 (2.9%) |
| COPD, AF | CKD | 1.52 | 17 (1.8%) |
| CKD, COPD | Anemia | 1.51 | 18 (1.9%) |
| AF | CKD | 1.49 | 60 (6.5%) |
| Osteoporosis | COPD | 1.49 | 18 (1.9%) |
| CVD, COPD | Diabetes | 1.48 | 11 (1.2%) |
| CKD | Anemia | 1.47 | 71 (7.7%) |
| Anemia | CKD | 1.47 | 71 (7.7%) |
| Diabetes, anemia | COPD | 1.46 | 15 (1.6%) |
| HF, AF | CKD | 1.46 | 18 (1.9%) |
| CKD, COPD | Diabetes | 1.46 | 15 (1.6%) |
| Diabetes, CAD | COPD | 1.43 | 13 (1.4%) |
| CAD, AF | CKD | 1.40 | 12 (1.3%) |
| CKD, CAD | Anemia | 1.40 | 10 (1.1%) |
| CAD | COPD | 1.37 | 44 (4.8%) |
| CAD, HF | COPD | 1.36 | 12 (1.3%) |
| Diabetes, CVD | COPD | 1.35 | 11 (1.2%) |
| Diabetes, CKD | Anemia | 1.34 | 16 (1.7%) |
| Diabetes, CKD | COPD | 1.29 | 15 (1.6%) |
|  |  |  |  |
