# Supplementary material for: Interactions between patterns of multimorbidity and functional status among hospitalized older patients: a novel approach using cluster analysis and association rule mining
Source: J Transl Med. 2024 Jul 18;22:669. doi: 10.1186/s12967-024-05444-9 (PMC11264579; doi:10.1186/s12967-024-05444-9)
Supplement: Supplementary file 3 — Additional file 3: Table S3. List of the 73 association rules showing disease relationships in the mildly dependent group, cluster 1. AF: atrial fibrillation; CAD: coronary artery disease/ischemic cardiomyopathy; CKD: chronic kidney disease; COPD: chronic obstructive pulmonary disease; CVD: cerebrovascular disease; HF: heart failure. [file 12967_2024_5444_MOESM3_ESM.docx]

| **Antecedent diseases** | **Consequent disease** | **Lift** | **Support** |
| --- | --- | --- | --- |
| COPD, AF, Anemia | CKD | 2.64 | 9 (1.2%) |
| CKD, COPD, AF | Anemia | 2.49 | 9 (1.2%) |
| CKD, AF, anemia | COPD | 2.35 | 9 (1.2%) |
| Diabetes, AF | HF | 2.18 | 13 (1.7%) |
| Diabetes, COPD | CAD | 2.18 | 15 (2.0%) |
| CKD, COPD, anemia | AF | 2.16 | 9 (1.2%) |
| CAD, AF | HF | 2.14 | 9 (1.2%) |
| CAD, AF | COPD | 2.09 | 12 (1.6%) |
| Anemia, prostate hypertrophy | CKD | 2.01 | 8 (1.1%) |
| COPD, HF | AF | 1.92 | 16 (2.1%) |
| Diabetes, COPD | HF | 1.90 | 13 (1.7%) |
| COPD, AF | HF | 1.86 | 16 (2.1%) |
| CKD, HF | CAD | 1.84 | 15 (2.0%) |
| CVD, COPD | CAD | 1.83 | 10 (1.3%) |
| CAD, anemia | CKD | 1.76 | 15 (2.0%) |
| Diabetes, HF | AF | 1.74 | 13 (1.7%) |
| Diabetes, CAD | COPD | 1.74 | 15 (2.0%) |
| Parkinson’s | CVD | 1.65 | 15 (2.0%) |
| Thyroid disorders | AF | 1.63 | 19 (2.5%) |
| CAD, COPD | Diabetes | 1.63 | 15 (2.0%) |
| COPD, HF | Diabetes | 1.60 | 13 (1.7%) |
| Diabetes, HF | COPD | 1.60 | 13 (1.7%) |
| Diabetes, AF | COPD | 1.60 | 13 (1.7%) |
| HF | AF | 1.56 | 45 (6.0%) |
| CVD, HF | Diabetes | 1.56 | 9 (1.2%) |
| CVD, CAD | Diabetes | 1.56 | 11 (1.5%) |
| Diabetes, HF | CKD | 1.55 | 15 (2.0%) |
| CKD, HF | Diabetes | 1.52 | 15 (2.0%) |
| Diabetes, COPD | AF | 1.52 | 13 (1.7%) |
| CVD, COPD | Diabetes | 1.51 | 10 (1.3%) |
| COPD, anemia | CKD | 1.49 | 19 (2.5%) |
| HF, AF | COPD | 1.48 | 16 (2.1%) |
| Prostate hypertrophy | CKD | 1.48 | 24 (3.2%) |
| CAD, HF | CKD | 1.47 | 15 (2.0%) |
| CVD, dementia | Diabetes | 1.46 | 10 (1.3%) |
| Cancer | Anemia | 1.45 | 23 (3.1%) |
| AF, anemia | CKD | 1.45 | 16 (2.1%) |
| Dementia, COPD | AF | 1.41 | 9 (1.2%) |
| Diabetes, anemia | CKD | 1.41 | 12 (1.6%) |
| CKD, COPD | Anemia | 1.41 | 19 (2.5%) |
| CVD, CKD | Diabetes | 1.40 | 9 (1.2%) |
| CKD, prostate hypertrophy | Anemia | 1.38 | 8 (1.1%) |
| HF, anemia | CKD | 1.38 | 9 (1.2%) |
| Diabetes, CAD | CKD | 1.37 | 14 (1.9%) |
| Diabetes, anemia | AF | 1.37 | 9 (1.2%) |
| CAD | CKD | 1.36 | 51 (6.8%) |
| CAD | COPD | 1.36 | 43 (5.8%) |
| CKD, AF | Anemia | 1.35 | 16 (2.1%) |
| Diabetes, AF | CKD | 1.35 | 13 (1.7%) |
| Dementia, AF | COPD | 1.34 | 9 (1.2%) |
| Dementia, COPD | CKD | 1.34 | 11 (1.5%) |
| Anemia | CKD | 1.33 | 68 (9.1%) |
| CKD | Anemia | 1.33 | 68 (9.1%) |
| Prostate hypertrophy | COPD | 1.32 | 18 (2.4%) |
| CAD, COPD | CKD | 1.32 | 16 (2.1%) |
| CKD, CAD | COPD | 1.31 | 16 (2.1%) |
| Dementia, anemia | CKD | 1.29 | 11 (1.5%) |
| AF, anemia | COPD | 1.28 | 12 (1.6%) |
| CKD, AF | COPD | 1.28 | 15 (2.0%) |
| Dementia, CKD | COPD | 1.27 | 11 (1.5%) |
| CAD, HF | COPD | 1.27 | 11 (1.5%) |
| Dementia, CKD | Anemia | 1.27 | 11 (1.5%) |
| Diabetes, COPD | CKD | 1.26 | 14 (1.9%) |
| CVD, CAD | COPD | 1.26 | 10 (1.3%) |
| CAD, anemia | COPD | 1.25 | 9 (1.2%) |
| HF | CKD | 1.24 | 46 (6.2%) |
| Diabetes | CKD | 1.23 | 56 (7.5%) |
| Prostate hypertrophy | CVD | 1.22 | 18 (2.4%) |
| COPD, HF | CKD | 1.20 | 13 (1.7%) |
| Diabetes, CAD | CVD | 1.18 | 11 (1.1%) |
| CAD, AF | CKD | 1.17 | 8 (1.1%) |
| COPD | CKD | 1.10 | 56 (7.5%) |
| COPD, AF | CKD | 1.08 | 15 (2.0%) |
