# Supplementary material for: Interactions between patterns of multimorbidity and functional status among hospitalized older patients: a novel approach using cluster analysis and association rule mining
Source: J Transl Med. 2024 Jul 18;22:669. doi: 10.1186/s12967-024-05444-9 (PMC11264579; doi:10.1186/s12967-024-05444-9)
Supplement: Supplementary file 4 — Additional file 4: Table S4. List of the association rules showing disease relationships in cluster 1 and 2 of the moderately-severely dependent group. AF: atrial fibrillation; CAD: coronary artery disease/ischemic cardiomyopathy; CKD: chronic kidney disease; COPD: chronic obstructive pulmonary disease; CVD: cerebrovascular disease; HF: heart failure. [file 12967_2024_5444_MOESM4_ESM.docx]

| **Antecedent diseases** | **Consequent disease** | **Lift** | **Support** |
| --- | --- | --- | --- |
| ***Cluster 1, rules=13*** |  |  |  |
| CAD, HF | AF | 1.97 | 17 (1.0%) |
| Thyroid disorder | AF | 1.75 | 32 (1.9%) |
| CVD, CAD | AF | 1.55 | 20 (1.2%) |
| COPD, HF | AF | 1.51 | 17 (1.0%) |
| CAD | AF | 1.50 | 58 (3.4%) |
| HF | AF | 1.49 | 76 (4.5%) |
| Parkinson’s | CVD | 1.46 | 58 (3.4%) |
| Thyroid Dysfunction | CVD | 1.21 | 31 (1.8%) |
| CAD, AF | CVD | 1.20 | 20 (1.2%) |
| CAD | CVD | 1.16 | 63 (3.7%) |
| Diabetes, AF | CVD | 1.14 | 19 (1.1%) |
| AF | CVD | 1.11 | 111 (6.5%) |
| Diabetes | CVD | 1.08 | 91 (5.4%) |
| ***Cluster 2, rules=23*** |  |  |  |
| Hypertension, anemia | CKD | 1.43 | 78 (4.6%) |
| Dementia, CKD, anemia | Hypertension | 1.42 | 35 (2.1%) |
| Dementia, anemia | Hypertension | 1.39 | 116 (6.8%) |
| Dementia, CKD | Hypertension | 1.38 | 99 (5.8%) |
| CKD, hypertension | Anemia | 1.38 | 78 (4.6%) |
| Anemia | CKD | 1.37 | 152 (9.0%) |
| CKD | Anemia | 1.37 | 152 (9.0%) |
| Dementia, CKD, hypertension | Anemia | 1.34 | 35 (2.1%) |
| Dementia, hypertension | Anemia | 1.31 | 116 (6.8%) |
| Dementia, CKD | Anemia | 1.30 | 62 (3.7%) |
| CKD, anemia | Hypertension | 1.29 | 78 (4.6%) |
| Hypertension | CKD | 1.28 | 214 (12.6%) |
| CKD | Hypertension | 1.28 | 214 (12.6%) |
| Hypertension, anemia | Dementia | 1.24 | 116 (6.8%) |
| Hypertension | Anemia | 1.23 | 220 (13.0%) |
| Anemia | Hypertension | 1.23 | 220 (13.0%) |
| Dementia, hypertension, anemia | CKD | 1.22 | 35 (2.1%) |
| Hypertension | Dementia | 1.17 | 335 (19.7%) |
| Dementia | Hypertension | 1.17 | 335 (19.7%) |
| Anemia | Dementia | 1.10 | 210 (12.4%) |
| CKD, hypertension | Dementia | 1.09 | 99 (5.8%) |
| CKD, hypertension, anemia | Dementia | 1.06 | 35 (2.1%) |
| CKD | Dementia | 1.01 | 180 (10.6%) |
