# Supplementary material for: Interactions between patterns of multimorbidity and functional status among hospitalized older patients: a novel approach using cluster analysis and association rule mining
Source: J Transl Med. 2024 Jul 18;22:669. doi: 10.1186/s12967-024-05444-9 (PMC11264579; doi:10.1186/s12967-024-05444-9)
Supplement: Supplementary file 5 — Additional file 5: Table S5. Differences in the prevalence of chronic diseases and ADL impairment between men and women. ADL: activity of daily living; AF: atrial fibrillation; CAD: coronary artery disease/ischemic cardiomyopathy; CKD: chronic kidney disease; COPD: chronic obstructive pulmonary disease; CVD: cerebrovascular disease; HF: heart failure. [file 12967_2024_5444_MOESM5_ESM.docx]

|  | | Men (n=1,354) | Women (n=2,012) | *p* |
| --- | --- | --- | --- | --- |
| Hypertension | | 583 (43.1) | 898 (44.6) | 0.386 |
| Dementia | | 285 (21.0) | 634 (31.5) | <0.001 |
| CVD | | 371 (27.4) | 479 (23.8) | 0.021 |
| CKD | | 400 (29.5) | 435 (21.6) | <0.001 |
| Anemia | | 242 (25.2) | 506 (25.1) | 0.975 |
| COPD | | 337 (24.9) | 362 (18.0) | <0.001 |
| AF | | 289 (21.3) | 406 (20.2) | 0.438 |
| Diabetes | | 279 (20.6) | 366 (18.2) | 0.089 |
| HF | | 520 (38.4) | 854 (42.4) | 0.021 |
| CAD | | 222 (16.4) | 237 (11.8) | <0.001 |
| Cancer | | 154 (11.4) | 143 (7.1) | <0.001 |
| Parkinson’s disease | | 94 (6.9) | 90 (4.5) | 0.003 |
| Thyroid dysfunction | | 45 (3.3) | 159 (7.9) | <0.001 |
| Osteoporosis | | 31 (2.3) | 159 (7.9) | <0.001 |
| ADL impairment | |  |  | <0.001 |
|  | No | 467 (34.5) | 456 (22.7) |  |
|  | Mild | 303 (22.4) | 444 (22.1) |  |
|  | Moderate-severe | 584 (43.1) | 1,112 (55.2) |  |
