# Supplementary figures and images for: Interactions between patterns of multimorbidity and functional status among hospitalized older patients: a novel approach using cluster analysis and association rule mining
Source: J Transl Med. 2024 Jul 18;22:669. doi: 10.1186/s12967-024-05444-9 (PMC11264579; doi:10.1186/s12967-024-05444-9)

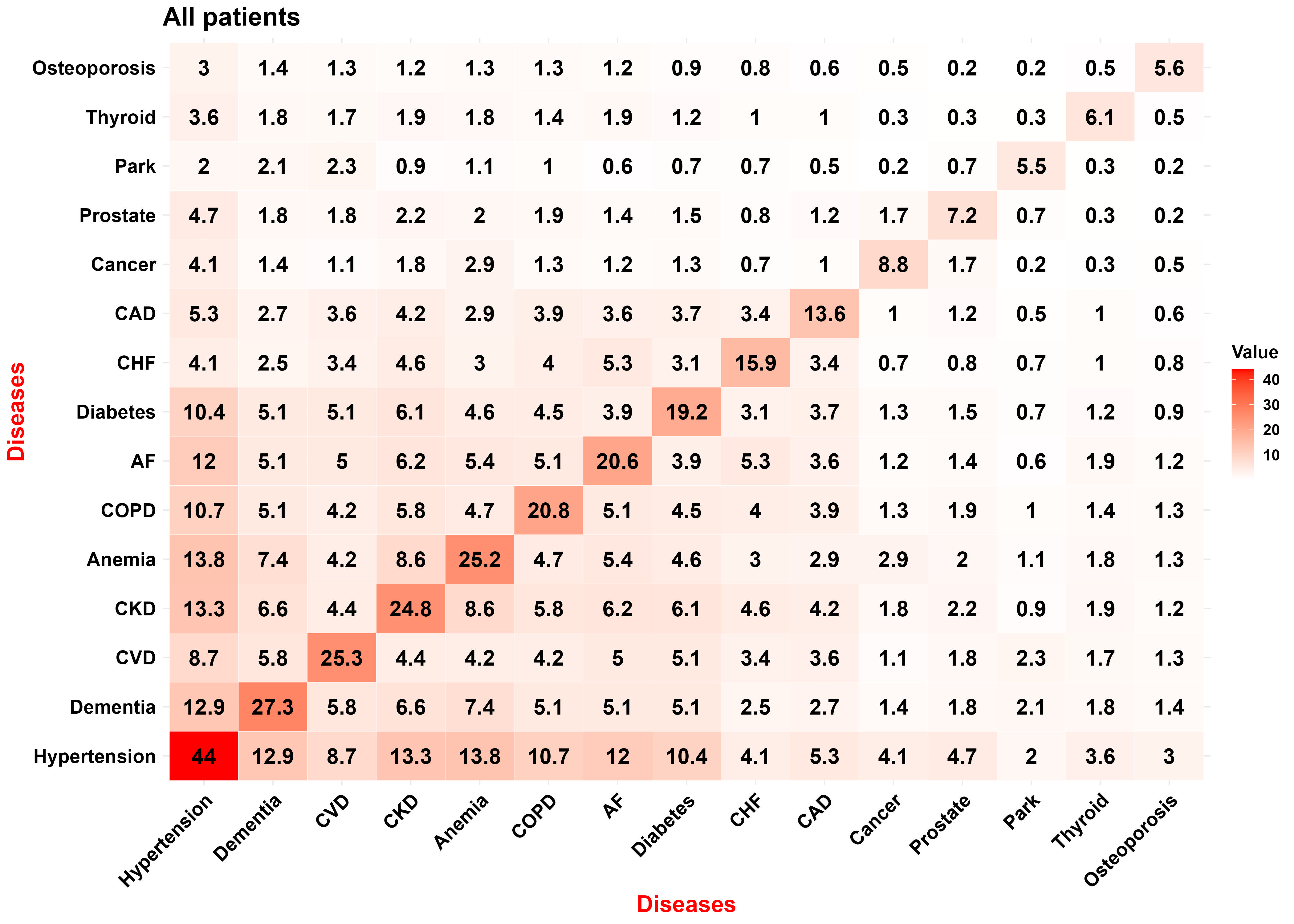

Supplement: Supplementary file 6 — Additional file 6: Figure S1. Heatmap representation of the co-occurrence of disease pairs in the whole study population. Each cell in the heatmap corresponds to a unique combination of diseases, with the color intensity indicating the strength of co-occurrence. Darker shades represent higher joint prevalence, highlighting disease pairs that commonly occur together. [file 12967_2024_5444_MOESM6_ESM.jpg]

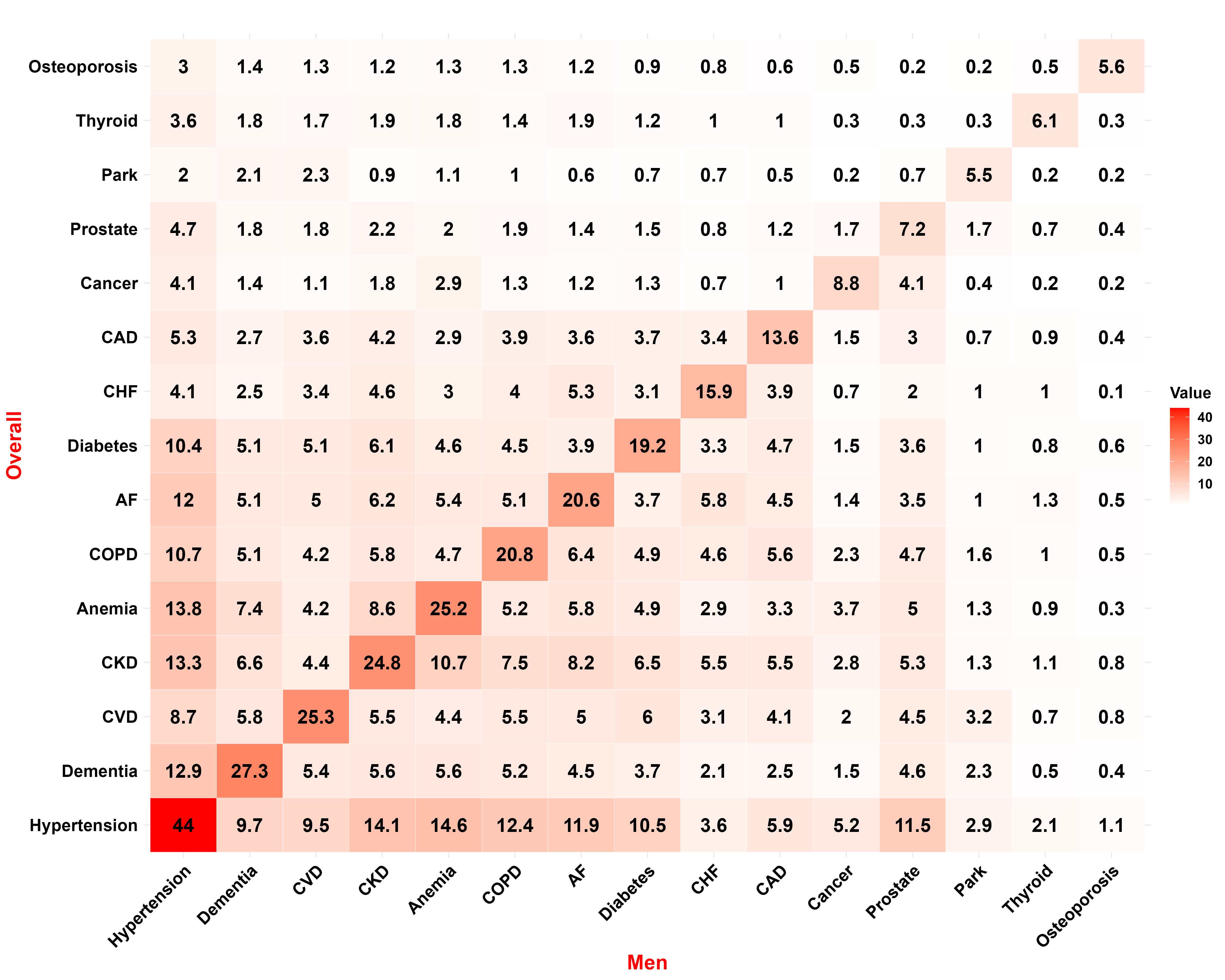

Supplement: Supplementary file 7 — Additional file 7: Figure S2. Heatmap representation of the co-occurrence of disease pairs in the whole study population (upper part) and among men (lower part). Each cell in the heatmap corresponds to a unique combination of diseases, with the color intensity indicating the strength of co-occurrence. Darker shades represent higher joint prevalence, highlighting disease pairs that commonly occur together. [file 12967_2024_5444_MOESM7_ESM.jpg]

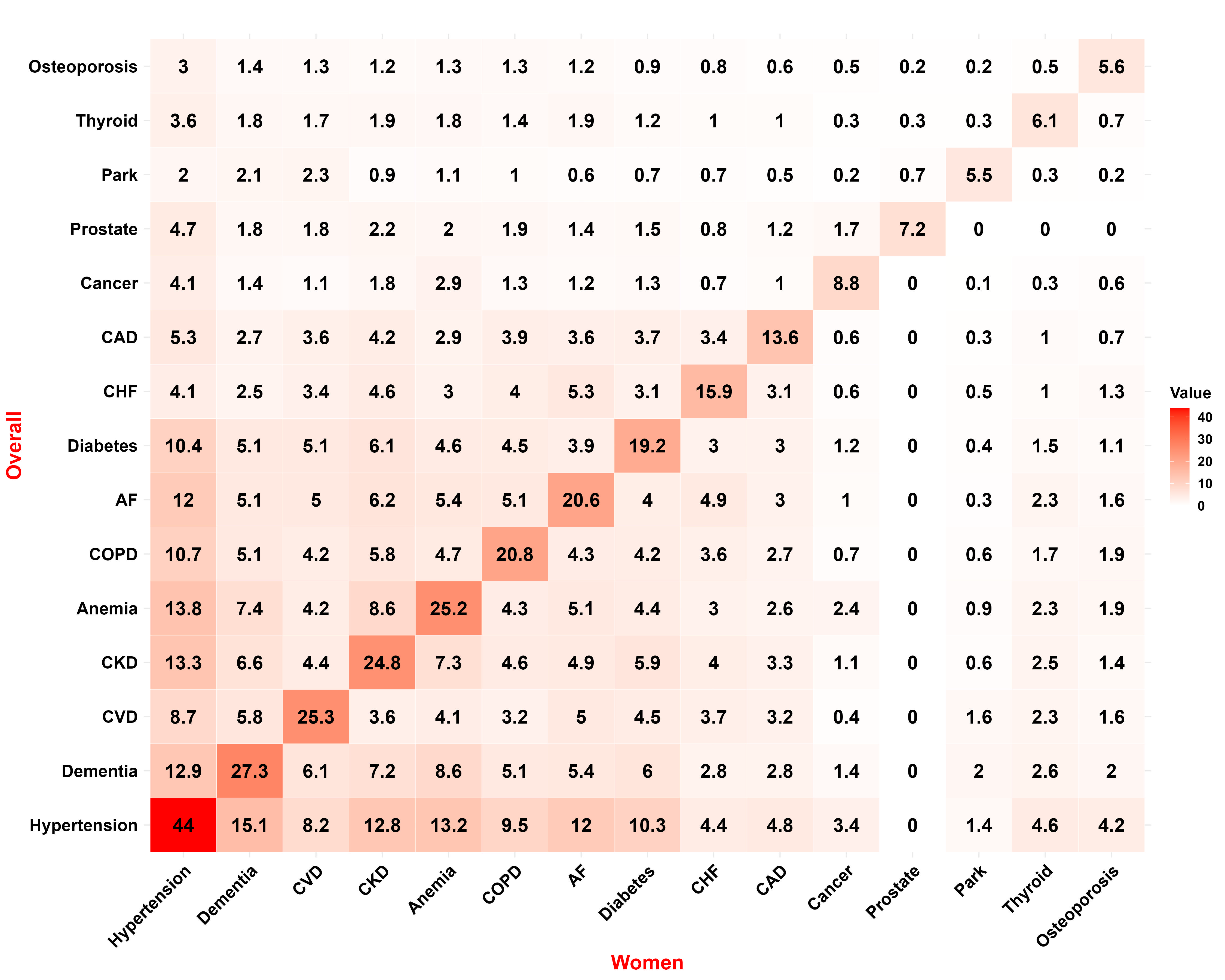

Supplement: Supplementary file 8 — Additional file 8: Figure S3. Heatmap showing the co-occurrence of disease pairs in the whole study population (upper part) and among women (lower part). Each cell in the heatmap corresponds to a unique combination of diseases, with the color intensity indicating the strength of co-occurrence. Darker shades represent higher joint prevalence, highlighting disease pairs that commonly occur together. [file 12967_2024_5444_MOESM8_ESM.jpg]

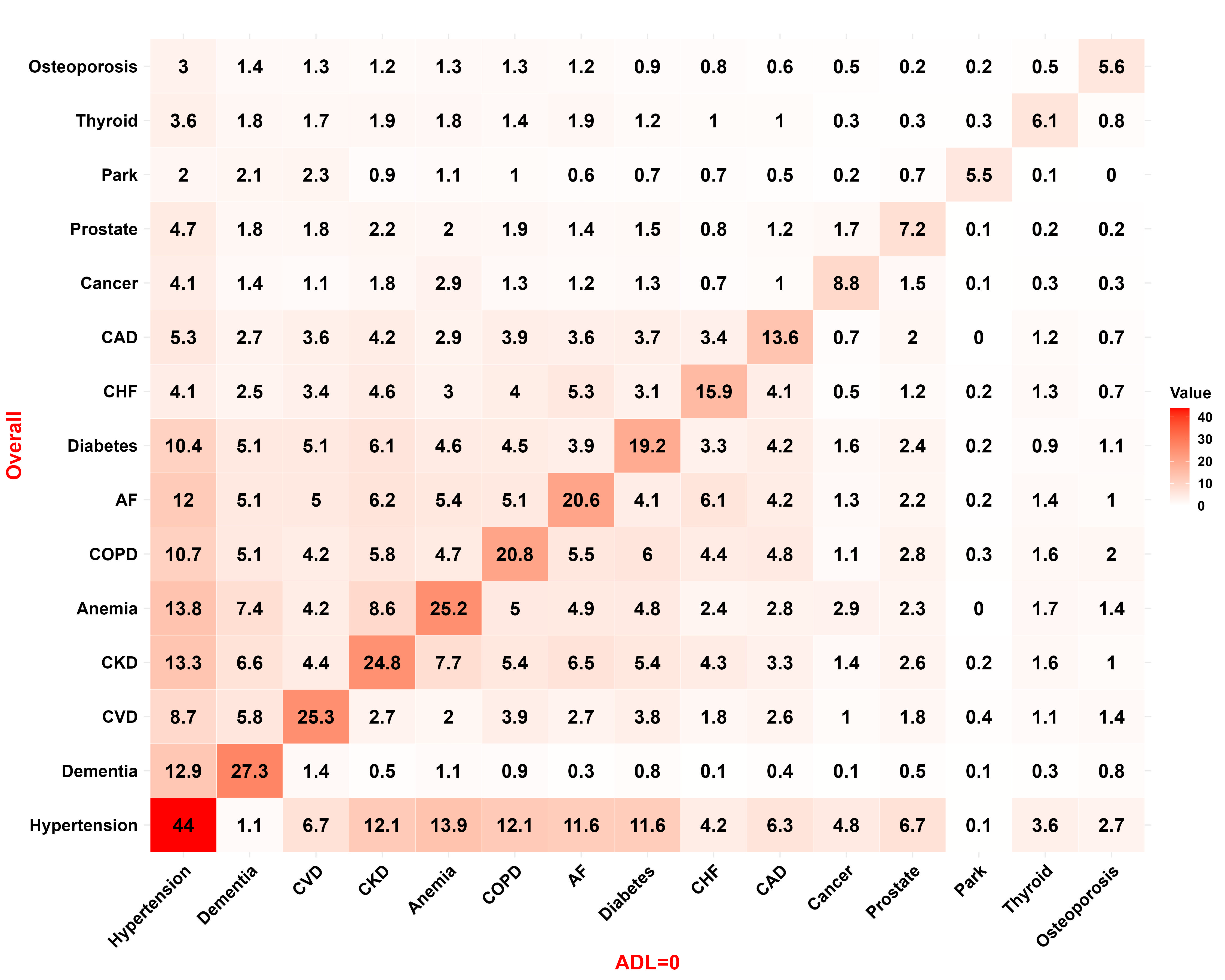

Supplement: Supplementary file 9 — Additional file 9: Figure S4. Heatmap showing the co-occurrence of disease pairs in the whole study population (upper part) and among functionally independent (ADL = 0) patients (lower part) Each cell in the heatmap corresponds to a unique combination of diseases, with the color intensity indicating the strength of co-occurrence. Darker shades represent higher joint prevalence, highlighting disease pairs that commonly occur together. [file 12967_2024_5444_MOESM9_ESM.jpg]

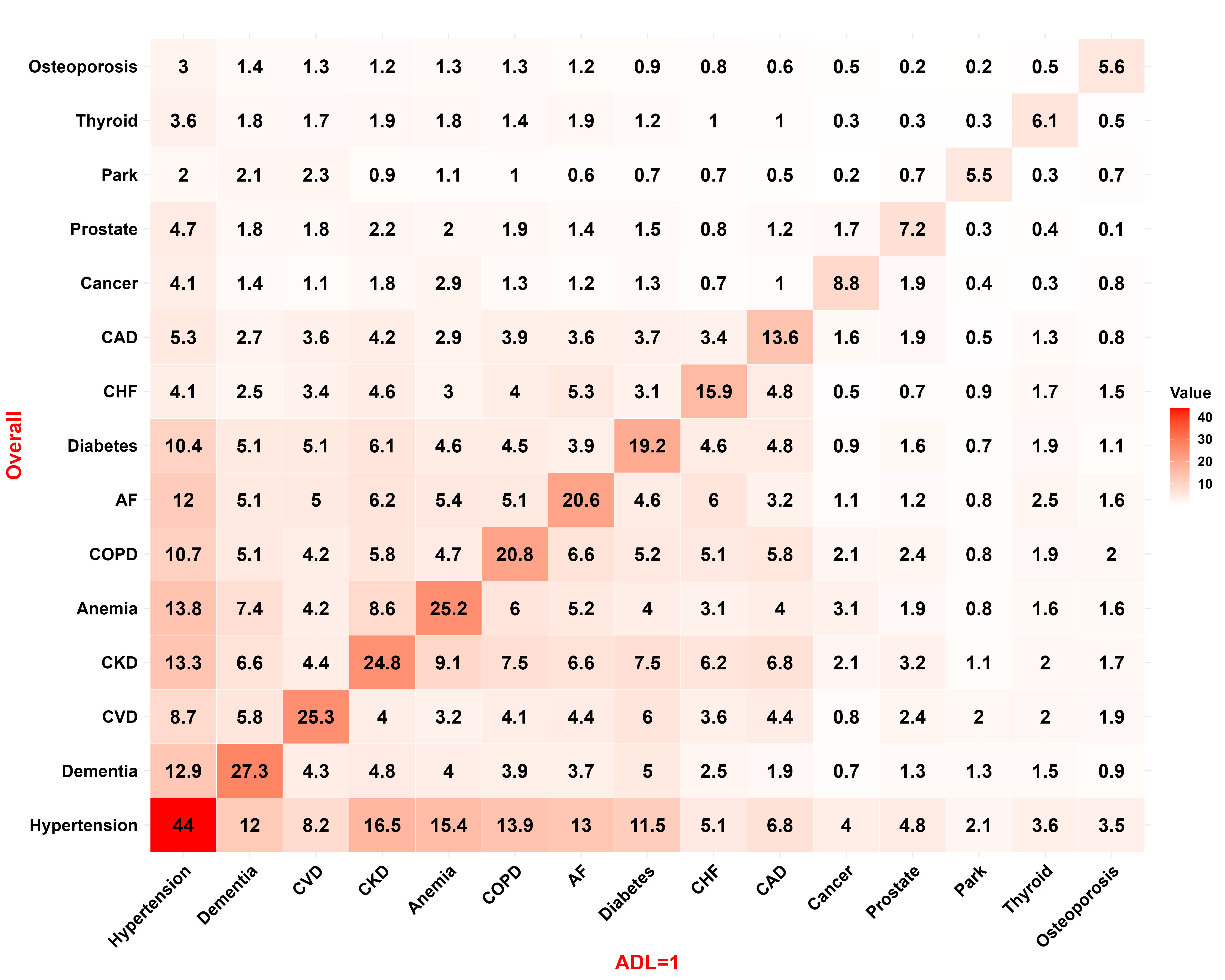

Supplement: Supplementary file 10 — Additional file 10: Figure S5. Heatmap showing the co-occurrence of disease pairs in the whole study population (upper part) and among mildly functionally dependent (ADL = 1) patients (lower part). Each cell in the heatmap corresponds to a unique combination of diseases, with the color intensity indicating the strength of co-occurrence. Darker shades represent higher joint prevalence, highlighting disease pairs that commonly occur together. [file 12967_2024_5444_MOESM10_ESM.jpg]

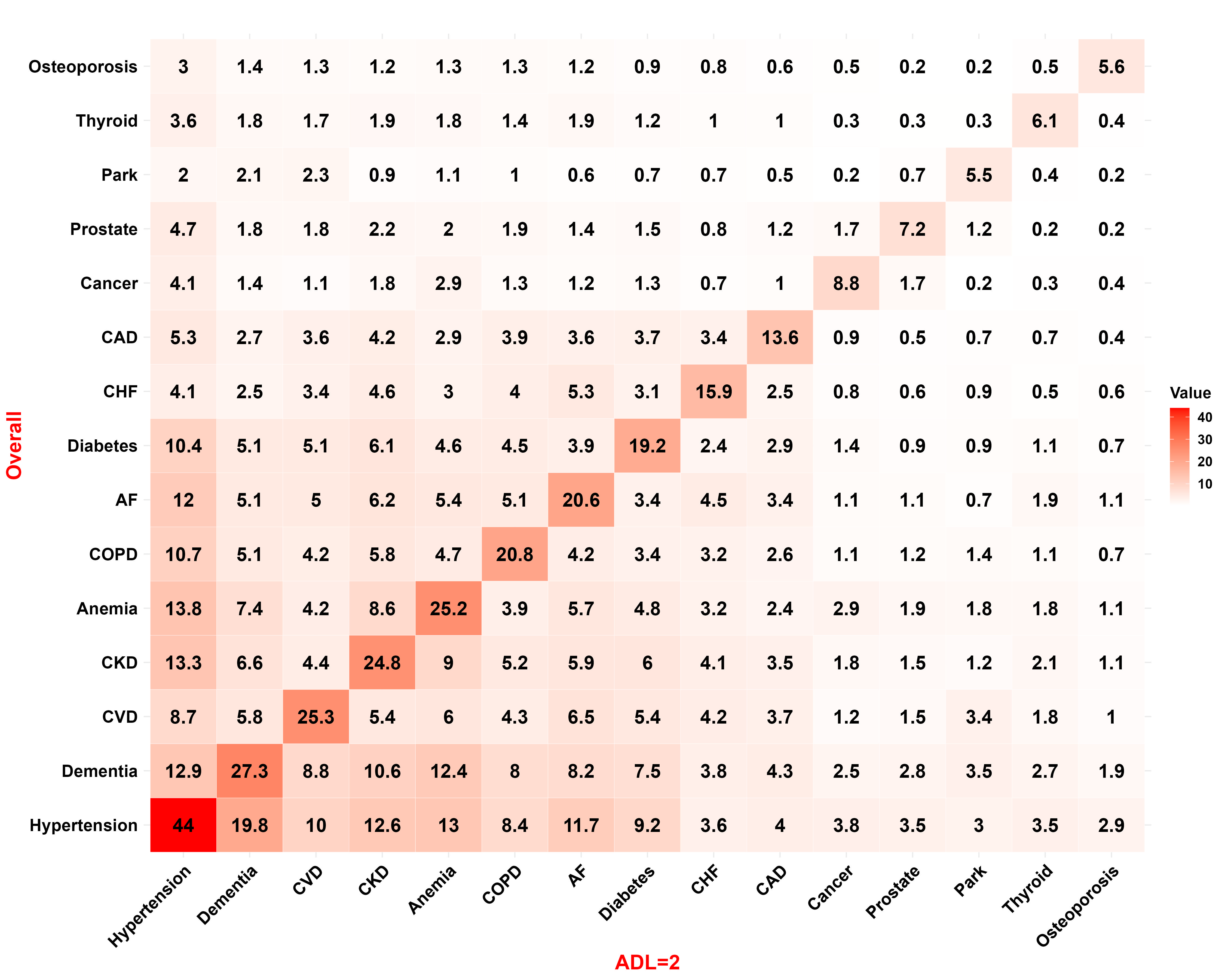

Supplement: Supplementary file 11 — Additional file 11: Figure S6. Heatmap showing the co-occurrence of disease pairs in the whole study population (upper part) and among moderately-severely functionally dependent (ADL = 2) patients (lower part). Each cell in the heatmap corresponds to a unique combination of diseases, with the color intensity indicating the strength of co-occurrence. Darker shades represent higher joint prevalence, highlighting disease pairs that commonly occur together. [file 12967_2024_5444_MOESM11_ESM.jpg]

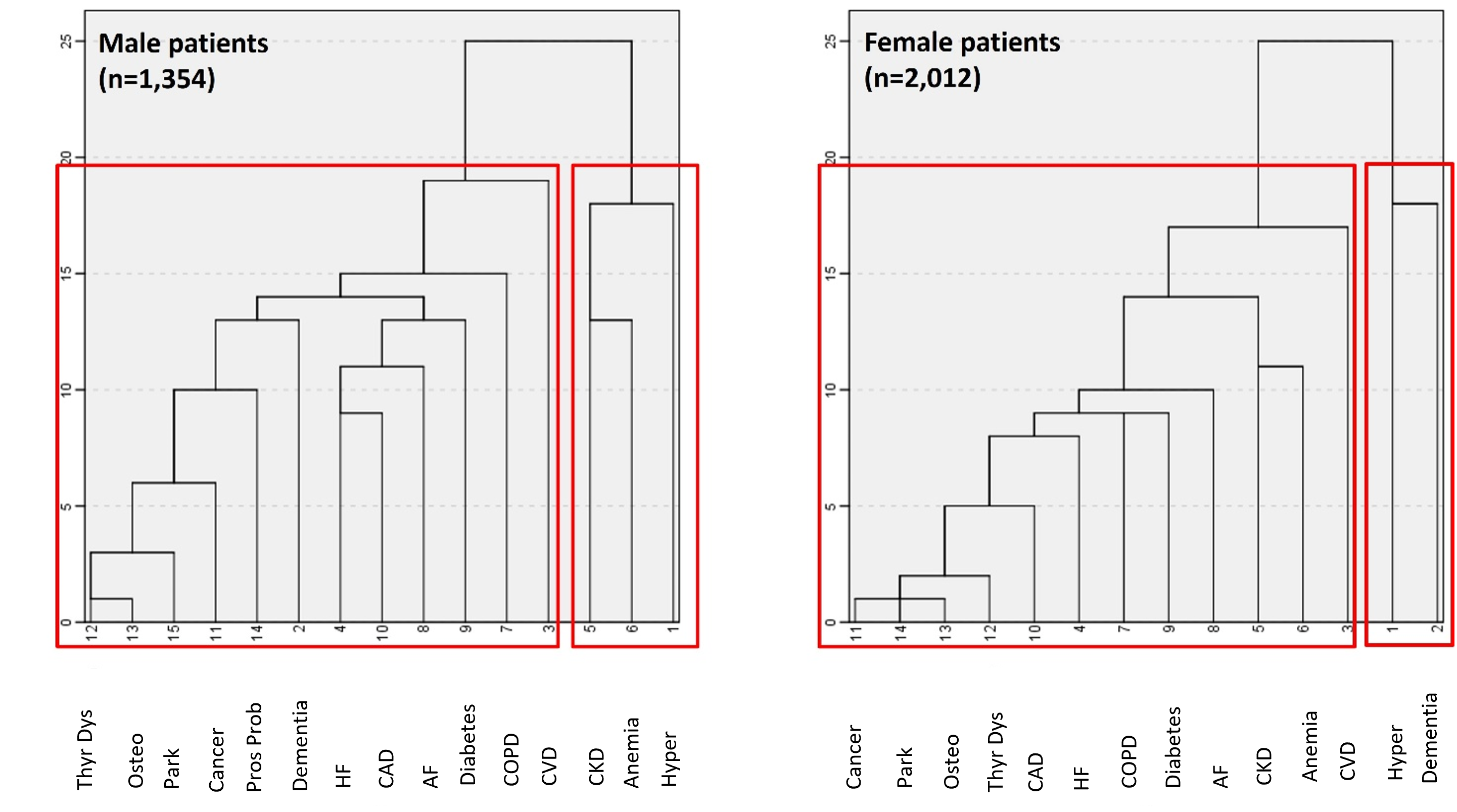

Supplement: Supplementary file 12 — Additional file 12: Figure S7. Hierarchical cluster analysis showing MM patterns in women and men in the whole population. AF: atrial fibrillation; CAD: coronary artery disease/ischemic cardiomyopathy; CKD: chronic kidney disease; COPD: chronic obstructive pulmonary disease; CVD: cerebrovascular disease; HF: heart failure. [file 12967_2024_5444_MOESM12_ESM.png]

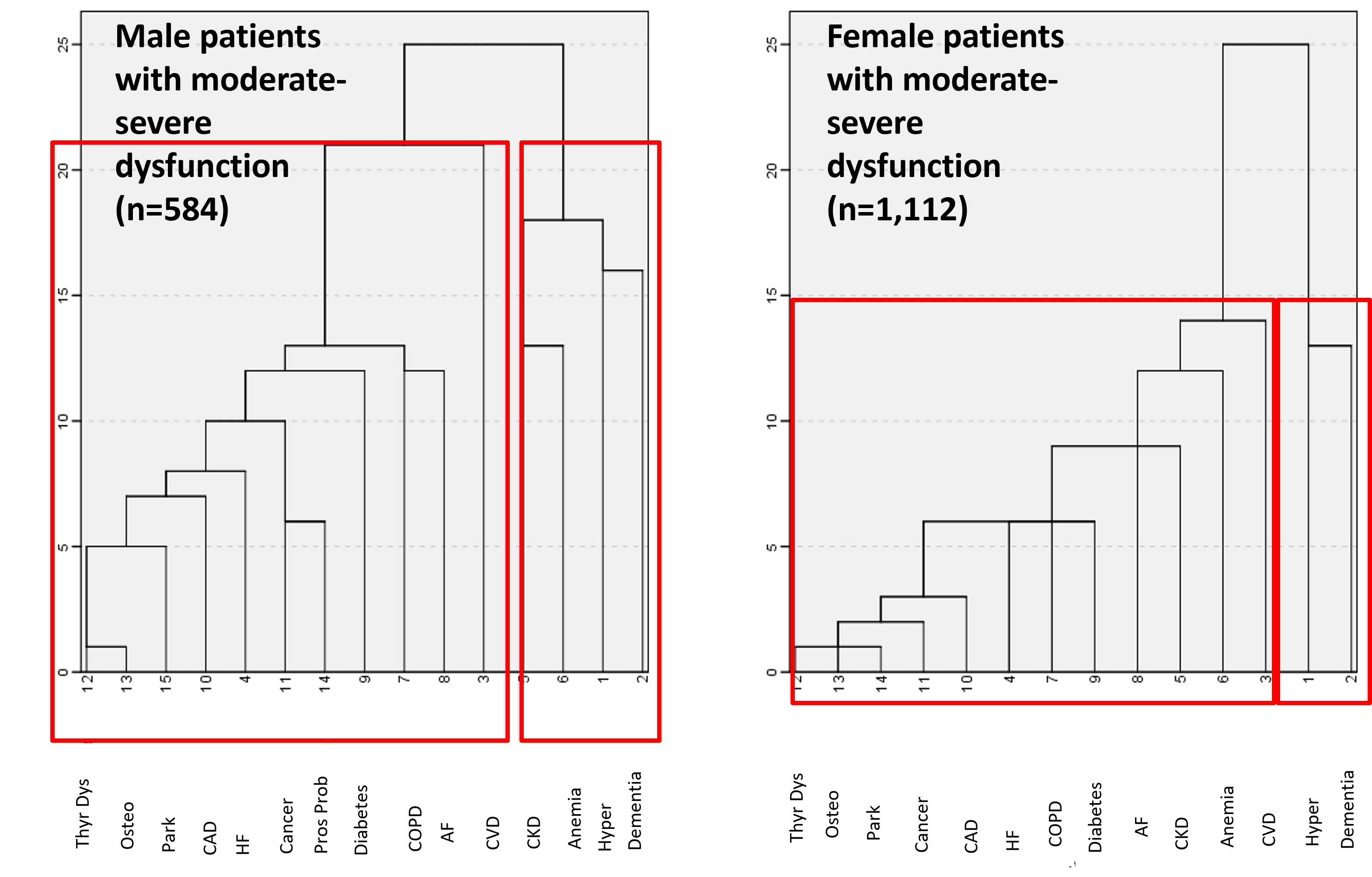

Supplement: Supplementary file 13 — Additional file 13: Figure S8. Hierarchical cluster analysis showing MM patterns in women and men in the moderately-severely dependent group. AF: atrial fibrillation; CAD: coronary artery disease/ischemic cardiomyopathy; CKD: chronic kidney disease; COPD: chronic obstructive pulmonary disease; CVD: cerebrovascular disease; HF: heart failure. [file 12967_2024_5444_MOESM13_ESM.png]
